# Between division and connection: a qualitative study of the impact of COVID-19 restrictions on social relationships in the United Kingdom

**DOI:** 10.12688/wellcomeopenres.17452.1

**Published:** 2022-01-06

**Authors:** Mira Leonie Schneiders, Constance R.S. Mackworth-Young, Phaik Yeong Cheah

**Affiliations:** 1 Mahidol Oxford Tropical Medicine Research Unit, Faculty of Tropical Medicine, Mahidol University, Bangkok, 10400, Thailand; 2 Centre for Tropical Medicine & Global Health, Nuffield Department of Medicine, University of Oxford, Oxford, UK; 3 Ethox Centre, Big Data Institute, Nuffield Department of Population Health, University of Oxford, Oxford, UK; 4 Department of Global Health and Development, London School of Hygiene and Tropical Medicine, London, UK; 5 The SoNAR-Global Network, Mahidol University, Bangkok, 10400, Thailand

**Keywords:** COVID-19, lived experience, social support, relationships, qualitative, well-being, Community and Public Health

## Abstract

**Background**: The first national COVID-19 lockdown in the United Kingdom between March to July 2020 resulted in sudden and unprecedented disruptions to daily life. This study sought to understand the impact of COVID-19 non-pharmaceutical interventions (NPIs), such as social distancing and quarantine, on people’s lived experiences, focusing on social connections and relationships.

**Methods**: Data were generated through 20 in-depth online and telephone interviews, conducted between May and July 2020, and analysed using thematic analysis informed by an ecological framework.

**Results**: Findings show that the use of NPIs impacted social relationships and sociality at every level, disrupting participant’s sense of self; relationships with their partners, household members, neighbours, and communities; and polarising social and political views. However, experiences of personal meaning-making and reflection, and greater social connectedness, solidarity, and compassion – despite physical distance – were also common.

**Conclusions**: Participant’s lived experiences of the first UK lockdown underscore the interconnectedness of relationships at the individual, community and societal level and point towards the important role of trust, social cohesion, and connectedness in coping with pandemic stress and adversity. Where infectious disease prevention measures rupture sociality, support for social connection at every relational level is likely to help build resilience in light of ongoing COVID-19 restrictions.

## Introduction

Around the world, the SARS-CoV-2 (COVID-19) pandemic has resulted in the implementation of many non-pharmaceutical interventions (NPIs) to reduce viral transmission ( World Health Organization, 2019). In the United Kingdom (UK), the first COVID-19 case was recorded on 31 January 2020 and between April and May 2020, the UK was experiencing its first COVID-19 wave, peaking on 8 April 2020 with 1075 deaths among people who tested positive for COVID-19 within 28 days of death ( Office for National Statistics, 2021; UK Government COVID-19, 2021). The first UK nationwide lockdown was imposed on 23 March 2020, involving social distancing and isolation, quarantine, closure of schools and non-essential shops, services and workplaces, ban on public gathering and leaving home for non-essential reasons, as well as guidance on handwashing and respiratory hygiene ( National Health Service UK, 2020). When implemented at scale, these measures result in what is commonly known as a “lockdown”. While NPIs are evidenced to be effective at reducing COVID-19 transmission, mortality and healthcare demand ( Doung-Ngern *et al.*, 2020; Ferguson *et al.*, 2020; Flaxman *et al.*, 2020; Koo *et al.*, 2020), little social science research has been published on the psychosocial impact of lockdowns on the public ( Kittel *et al.*, 2020; Norton *et al.*, 2020a), despite this being identified as a global priority research area ( Holmes *et al.*, 2020; Norton *et al.*, 2020b; World Health Organization).

In the UK, a handful of studies have assessed public perceptions, behaviours and experiences of the national lockdown (e.g. ( Atchison *et al.*, 2021; Brooke & Clark, 2020; Denford *et al.*, 2020; Keyworth *et al.*, 2021; Williams *et al.*, 2020)) and coping strategies ( Ogueji *et al.*, 2021), pointing towards pervasive experiences of psychological, physical and economic challenges related to NPIs. A UK-based focus group study on public perceptions during the early stages of lockdown showed that social distancing and isolation led to widely perceived losses, including loss of income, social interactions and psychological wellbeing, concerns about the future, and negative perceptions of the government ( Williams *et al.*, 2020). Other UK studies have shown adverse effects of the first weeks of lockdown on mental health, particularly among certain groups (e.g. young people, women, socially disadvantaged groups, COVID-19 risk groups, people with pre-existing mental health problems) ( Jia *et al.*, 2020; O'Connor *et al.*, 2020), as well as an increase in suicidal ideation ( O'Connor *et al.*, 2020). Furthermore, a study comparing the impact of lockdown on the public in the UK, US, Australia and Norway found highest levels of emotional distress and loneliness, and poorer wellbeing and quality of life among UK residents ( Geirdal *et al.*, 2021). In international comparison, the UK was late to implement restrictions during the first wave of its epidemic and once implemented, the restrictions imposed during the first national lockdown were among the most stringent worldwide (‘Stringency index’ (SI) range: 0-100; with 100 indicating strictest public health response; UK SI over the study period: SI=69-76 ( Hale *et al.*, 2020; Osterrieder *et al.*, 2021).

Globally, social distancing measures have also been associated with high rates of depression and anxiety, leading to calls for policy makers to consider mental health problems an “ongoing and concurrent epidemic (i.e., a syndemic)” ( Castaldelli-Maia *et al.*, 2020). These pervasive findings highlight the importance of better understanding the mechanisms driving the association between social distancing and poor mental health. The central role of social connection in promoting health and wellbeing has been widely established ( Cohen, 2004; House *et al.*, 1988). Particularly in times of adversity and uncertainty, perceived social support can act as a buffer against stress ( Cohen, 2004), while perceived absence of social connections (loneliness) activates neurobiological mechanisms that contribute to poor mental and physical health, and early morbidity and mortality ( Cacioppo & Cacioppo, 2014). A recent survey conducted in Austria found that higher rates of social connectedness during the COVID-19 lockdown were associated with lower levels of stress, worry and fatigue, concluding that social connections can work to promote resilience and buffer against pandemic stress and poor mental and physical health ( Nitschke *et al.*, 2021).

As an infectious disease, transmitted through close human contact, measures to keep people apart are necessary to prevent transmission of COVID-19 ( Guo *et al.*, 2020; Wang *et al.*, 2020). Measures to control previous epidemics, such as Ebola, included breaking sociality and interrupting social intimacy, including restrictions on gatherings and preventing ritual washing of bodies at funerals ( Lipton, 2017). In Philip Strong’s classic essay on “epidemic psychology”, the disruption of social dynamics of human relationships caused by fatal epidemics is conceptualised as an “assault to public order”, as it forces a sudden reconstruction of the social and political world ( Strong, 1990, p. 249). Understanding the complex and wide-ranging impacts of COVID-19 lockdowns on people's lived experience therefore requires acknowledging the interactions and interdependencies of individuals with their households, families, communities and society at large — as these represent vital sources of sociality and social connection. Investigating the impact of NPIs to prevent COVID-19 on the disruption of sociality and relationships is thus a key focus of this study.

This socio-relational perspective on the lived experience of epidemic disease resonates with socio-ecological approaches, which postulate that human experiences and behaviours are best understood as an interplay between an individual and the broader social and environmental systems in which they live ( Bronfenbrenner, 1979; McLaren & Hawe, 2005). The original ecological framework ( Bronfenbrenner, 1979)—which has been widely applied in health research—originated from applying ecology to understand child development as a complex system of relationships influenced by the surrounding environment; including the immediate physical, familial and social environment, and the broader cultural, economic, political, historical and environmental context ( McLaren & Hawe, 2005). Ecological frameworks emphasise the interaction and reciprocal influence between the individual (intrapersonal; sense of self and self- perception), the interpersonal (micro- and mesosystem; including household, family, school and peers) and the structural context (exo- and macrosystem; including politics, social services, societal norms and attitudes) ( McLaren & Hawe, 2005). In this way, ecological frameworks object to the assumptions that individuals are independent actors who can be viewed in isolation from their social context, arguing instead that individual behaviours, experiences and outcomes — including health and wellbeing — are determined by interpersonal dynamics and wider systems within which individuals are embedded ( Bronfenbrenner, 1979; McLaren & Hawe, 2005).

While other qualitative studies have examined the lived experiences of being diagnosed with COVID-19 ( Missel *et al.*, 2021), the lived experience of living with COVID-19 NPIs has been scarcely investigated, particularly in the early stages of the epidemic, when NPIs were most unexpected and severe. Thus, this study sought to understand the impact of public health measures implemented as part of the first nationwide COVID-19 lockdown on the lived experiences of people living in the UK, with a particular focus on social connection and relationships. Drawing on an adaptive ecological framework, we analyse the impact of restrictive measures on the experience of relationships at five levels: i. individual, ii. household, iii. family and friends, iv. community and neighbours, and v. wider society and politics.

## Methods

### Study design

This study was nested within a mixed methods study (SEBCOV study ( Pan-Ngum *et al.*, 2020)) conducted across five countries (Thailand, Malaysia, Italy, Slovenia and the UK), aiming to understand the lived experiences and impact of NPIs during the COVID-19 pandemic. Here we report on the qualitative arm of the UK study, which included 20 in-depth interviews with UK residents conducted between 14 May and 23 July 2020. At the time of interviewing, all participants had experienced the strictest UK nationwide lockdown imposed between 23 March to 11 May 2020 (SI=69-76 ( Hale *et al.*, 2020; Osterrieder *et al*., 2021)), after which, varying degrees of NPIs remained in place across the four UK nations. The COREQ guidelines for conducting and reporting qualitative research were followed in this study ( Tong *et al.*, 2007). 

### Participant selection

Participants were recruited through pre-existing research and organisational social media channels (Facebook, Twitter), including targeted Facebook advertisements. Additionally, all participants who took part in the quantitative arm of the SEBCOV online survey ( Osterrieder *et al.*, 2021) were provided with a link to register their interest in the qualitative arm of the study. Finally, targeted emails were sent to five UK community organisations to support advertisement among their members, with the aim of achieving greater demographic and socio-economic diversity within the sample.

Individuals were provided with information about the study and invited to register their interest via a short online recruitment survey, which included questions on six socio-demographic characteristics: age, gender, number of household members, occupation, level of education and self-perceived COVID-19 risk. Among 156 individuals who registered their interest online, 40 individuals were selected to achieve maximum variation based on those six socio-demographic characteristics and invited to participate in the study via email. Of these, 22 responded, with two dropping out prior to the interview without stating a reason, resulting in 20 interviews being conducted. Data collection continued until thematic saturation, the point at which no significant new themes emerged ( Guest *et al.*, 2006). Participants were briefed about the aims and purpose of the research and consent was obtained in writing via email.

### Participant characteristics

Overall, 60% of all participants were female and 40% male, aged between 25-80 years ( Table 1). Twenty-five percent of participants had completed secondary and 75% tertiary education. Participant’s household size ranged between 1-7 people, with a majority occupying multi-person households and 30% living alone. Forty-one percent of participants perceived themselves to be at low-risk of COVID-19, 35% at medium-risk, 20% at high-risk. Participants worked in various professions, including 20% as healthcare workers. Twenty-five percent of participants were retired, and 10% unemployed.

**Table 1.** Participant characteristics.

Characteristic	United Kingdom
	n (%)
**Gender**	n=20
Female	12 (60.0)
Male	8 (40.0)
Other	0 (0.0)
**Age range**	
18–24	1 (5.0)
25–34	2 (10.0)
35–44	1 (5.0)
45–54	4 (20.0)
55–64	6 (30.0)
65–74	5 (25.0)
75–84	1 (5.0)
**Highest level of education**	
Primary	0 (0.0)
Secondary	5 (25.0)
Tertiary	15 (75.0)
**Number of household members**	
1	6 (30.0)
2	7 (35.0)
3	4 (20.0)
4	2 (10.0)
5	0 (0.0)
6	0 (0.0)
7	1 (5.0)
>7	0 (0.0)
**Overall self-perceived COVID-19 risk level**	
Low	9 (45.0)
Medium	7 (35.0)
High	4 (20.0)
**Occupation a**	
**1 Managers**	**2 (10.0)**
**2 Professionals**	**8 (40.0)**
**3 Technicians and Associate** **Professionals**	**3 (15.0)**
**Others (not specified in** **ISCO-08)**	**7 (35.0)**
Retired	5 (25.0)
Unemployed	2 (10.0)
**Occupational category**	
Healthcare worker	4 (20.0)
Non-healthcare worker	16 (80.0)

^a^ Occupations have been classified according to the International Standard Classification of Occupations 08 (ISCO-08) ( International Labour Organization, 2010).

### Data collection

The interview topic guide was developed based on the research aims of the SEBCOV study, which sought to understand the impact, perceptions and understanding of COVID-19 NPIs ( Pan-Ngum *et al.*, 2020). The interview topic guide, which was pilot tested and subsequently refined by the interviewer team, focused on three broad topics, namely: i. Experiences and perceptions of COVID-19 measures (i.e., social isolation, social distancing, travel restrictions and quarantine); ii. wellbeing and mental health; and iii. information, misinformation and rumours. Repeat interviews were not carried out. The interview topic guide can be found in the *Extended data*.

Interviews were conducted online via a videoconferencing platform (Microsoft Teams) or by telephone, based on participants preferences, with only the interviewer and participant present during the interview. The majority of interviews were audio recorded (not video recorded) and transcribed verbatim. For remaining interviews, summary scripts were written up immediately following the interview, based on detailed notes taken during the interview—a method which has been found to produce levels of detail comparable to interview transcription ( Halcomb & Davidson, 2006; Rutakumwa *et al.*, 2020). Interviews lasted between 40-70 minutes and were conducted at a time convenient to participants. Participants received a £10 electronic supermarket voucher as compensation. Interviews were conducted in English by MLS and CMY, both female postdoctoral researchers, resident in the UK, and trained and experienced in conducting qualitative research. The interviewers did not know participants prior to conducting the interview.

The qualitative dataset (consisting of audio recordings of interviews, interview transcripts and field notes) were processed and managed using Microsoft Word and NVivo. All raw data were stored in a password protected electronic file and were only accessible to study staff and authorized personnel. The study complied with the EU General Data Protection Regulation (GDPR).

### Data analysis

Data analysis was done using thematic analysis ( Braun & Clarke, 2006), focused on understanding “individual’s lived experiences within the world”\ ( Neubauer *et al.*, 2019). Data were collected iteratively, with insights emerging from each interview helping to inform subsequent interviews and analysis. Key themes emerging from interviews were iteratively discussed by the interviewers (MLS, CMY) during regular online meetings during and after data collection. Initially, MLS and CMY each applied open inductive coding to two interview transcripts. Following discussions in which emerging themes were grouped into overarching primary and secondary level codes, MLS and CMY developed a preliminary coding framework, based on an adaptive ecological framework ( Bronfenbrenner, 1979), involving five primary level codes (i. individual, ii. household, iii. family and friends, iv. community and neighbours, and v. wider society and politics). The coding framework was then tried and refined using indictive coding on two additional transcripts and subsequently applied to code all remaining transcripts (MLS coded 100% of interviews, CMY coded 25% of interviews). Data was managed using the qualitative data software NVivo (v12). The major themes identified—which reflect the five environmental systems with which an individual interacts according to the ecological framework—are presented in the results section below. Due to data protection regulations, participants could not be recontacted in order to provide feedback on the findings.

### Ethical approval

Ethics approval was granted by Oxford Tropical Research Ethics Committee (OxTREC, reference no.520-20).

## Results

Below we present findings under the five levels of the adapted ecological framework (i. individual, ii. household, iii. family and friends, iv. community and neighbours, and v. wider society and politics), providing supporting quotes from the interviews conducted.

### Impact on individuals: between fear, grief, and time for personal reflection

Participants experienced a range of emotions in response to the lockdown, the majority describing heightened fear, anxiety, and worries, including about contracting COVID-19, health of their loved ones, going outside and into shops, financial and job insecurity, and uncertainty about the future: *“I just felt… fear, of just thinking I don’t want to die breathing in broken glass, I don’t want that feeling. So, I went through all that horror, as everyone did, that absolute terror and trying to keep calm about it”* (male, 53 years, support worker). Some participants felt lonely and isolated, particularly those living alone, who struggled without “ *any physical touch whatsoever for 12 weeks”* (female, 63 years, retired).

Many participants also spoke about their grief over missing out on important milestones or significant life events during lockdown, which was perceived as a major disruption and disorientation to the sense of self and life plans. This included not being able to attend births, birthdays, family celebrations, holidays, reunions and funerals: *“these are all very basic things that we do in life that have been taken from us… [it’s] very sad”* (female, 63 years, retired). Grief and regret over lost time and missed milestones during lockdown was most poignantly expressed by older participants, some of whom felt they did not have long to live. Those who experienced a family member’s death during lockdown discussed how, without social contact, “ *it’s been extremely hard to grieve*” (female, 66 years, administrator). Several participants said that their struggles were exacerbated by the closure of support groups, such as religious and mental health groups, leaving them feeling unsupported:

*“I have ADHD… I also get support from alcoholics anonymous… I have attended therapy and classes, but they came to an end very quickly in the COVID situation. That was a very important source of support for me that went just like that... Without other sources of support, I’m really struggling*” (female, 48 years, support worker).

On a more positive note, some also discussed inadvertent positive impacts of the lockdown on their mental health and wellbeing, including having more time for loved ones, for oneself, and for engaging in hobbies and personal projects (e.g., taking online classes, learning new skills, gardening, DIY tasks, exercise, cooking): *“I have personally been feeling really quite good because I have just taken this opportunity to really look after myself… So from that point of view it's been really quite phenomenal. Having that time to just stop”* (female, 56 years, self-employed). The lockdown also prompted some to reflect on and re-evaluate what mattered most to them in life, including prioritising particular relationships, valuing nature and reconsidering work-life balance:

“ *The whole world works in excess. It makes you re-evaluate whether we need that. What I miss is just spending time with friends... And we work more to have a more expensive time with them, rather than more time with them. I have realised that money is not as important as I thought it was”* (male, 33 years, healthcare worker).

### Impact on household and intimate relationships: between closer bonds and ruptured relationships

Many participants said that they valued spending more time together as a family within their household: “ *it was a really good opportunity for us just to be the three of us again, which hasn’t happened probably since preschool*” (female, 56 years, self-employed). This was particularly pertinent for families with new-borns, who could spend more time together as an intimate family. However, many parents and carers attempting to juggle work and home-schooling reported struggling, feeling strained and worrying about their children who were “ *missing their friends and… doing lots of schoolwork*” (female, 46, finance officer), despite some saying that “ *the school [was] great at sending resources*” (female, 59 years, unemployed). This led to some increasingly valuing multigenerational support, which was viewed as having multiple benefits for parents, children, and grandparents or other extended family members: “ *I felt I was supporting my daughter with [her] children… That helped me… that gave me a role in life*” (female, 71 years, freelance professional).

In terms of relationships with intimate partners, the described impact of COVID-19 and related measures on participants appeared to accelerate pre-existing trends in their relationships. For some, the lockdown led to significant rupture or strain in their relationship: “ *it shows the cracks in our relationship… we’ve been bickering more, and the ways we would have previously overcome difficulties – a nice dinner, a romantic break – aren’t possible now.”* (male, 33 years, healthcare worker). For others, the shared experience and increased time spent together, led to their “ *relationship becom[ing] closer*” (female, 59 years, unemployed). Some said that the support of their partner was critical to overcoming challenges in the lockdown period: “ *my wife and daughter… they pulled me out of it in the end, the depression*” (male, 72 years, retired). Others, without existing partners, discussed the challenges of how “ *online dating came to a halt*” (female, 63 years, retired).

### Impact on family and friends: between virtual connection and a longing for physical touch

Separation from family was described as the most difficult lockdown experience by many participants: “ *I haven’t seen my parents for nearly six months, that’s been quite tough*” (male, 55 years, IT professional). This was felt particularly strongly for separation from older relatives—“ *My dad is in his last few years of his life. I wouldn’t risk visiting him, as he is so vulnerable, but… I would so like to see him in his last few years*” (female, 48 years, support worker)—and for very young relatives: “ *the worst thing by far is not seeing the grandchildren… because they are really developing*” (female, 59 years, retired). Some expressed that the fear of COVID-19 increased care and concern among family members: “ *I was genuinely worried for my parents, and they were genuinely worried for me, for the first time… ever*” (male, 33 years, healthcare worker). Several participants described feeling helpless, worried and anxious about their loved ones mental and physical health or the risk of dying from COVID-19: “ *I kept thinking, well what if we don’t see each other again, every time I speak to him”* (male, 53 years, support worker).

Virtual connectivity was reported to have increased substantially, including with close family and friends, and more distant contacts, sometimes after months or years of disconnection. Participants described using virtual connectivity in creative ways to support family members during lockdown:

“ *Facetime or Skype, that has been brilliant... once a week we cook online. This week we did science, teaching [my granddaughter] about the heart... It's hard for the parents to entertain them for 12 hours a day... So, if they are online to us, it breaks the day up*.” (female, 59 years, retired).

However, participants noted that, although this was highly valued, it was only a partial replacement for physical connection, which many participants continued to yearn for:

“ *I have two children… and four grandchildren… but they are in England and I live in Wales. [There has been] lots of Zooming going on... it’s been really good. The only thing is when they go… well it's just so quiet suddenly [holding back tears].”* (female, 80 years, retired)

The importance of touch was repeatedly mentioned: “ *I haven’t physically touched another human being for nearly two months [crying], and I think that is probably one of the most difficult things, because we do so much by touch*” (female, 56 years, healthcare worker). The inability to touch or hug family during social distancing was perceived as particularly difficult: “ *the look on [my grandson’s] face like I was telling him ‘don’t come near me, don’t hug me’: it was horrible, that really did upset me*” (female, 63 years, retired).

For many, COVID-19 and related measures also created tensions with family and friends because of differences of opinion about how to interpret and navigate government measures:

“ *people in my life who I normally agree with on lots of things… see differently: what is legal is maybe not what people are comfortable with. And I think trying to navigate that has been quite difficult and I think quite hard to predict*.” (female, 24 years, healthcare worker)

For some, this led to tensions and ruptures in relationships with family and friends: “ *It's created this kind of rift and tension where they're not seeing each other because of the different attitudes they have towards the rules*” (female, 24 years, healthcare worker).

### Impact on community and neighbours: between solidarity and distrust

Many participants said that they had *“seen a lot of the best in humanity”* (female, 48 years, support worker) during the lockdown, emphasising a growing sense of solidarity, unity, neighbourly spirit and communities coming together: *“all the neighbours were definitely looking out for each other and that was really nice to see”* (male, 39 years, social worker). In their narratives of connectedness, some described hands-on efforts by local community support groups or friends:

*“A friend of mine, he has been physically… and socially isolated… and if he wants something, I go and leave it on his doorstep and if I need something then he leaves it with me... I see that happening a lot… we can actually really care for one another”* (female, 56 years, self-employed).

Others described virtual and trans-geographic support efforts, such as COVID-19 online forums, saying that *“even just reading [about] that has been quite positive for me”* (female, 63 years, retired). Several said that volunteering and helping others made them feel better: *“At least I can do something. And that makes me feel quite productive”* (female, 63 years, retired).

At the same time, many also discussed the *“fear of being in contact with other people”* (female, 63 years, retired) and feelings of distrust, judgement and tension within their communities: *“What I see in the community is fractured, people arguing, people feeling unsafe”* (female, 56 years, self-employed). These narratives of division were linked to *“frustrations… over the people who don’t adhere to social distancing”* (male, 65 years, retired) and worries about *“people who are ignoring the restrictions”* (female, 63 years, retired). Those describing themselves as *“rule keeper[s]”* said that *“when you see people breaking the rules, you actually start to be a bit judgemental about it”* (female, 59 years, retired). Consequently, many expressed a general sense of distrust towards others— *“I don’t trust other people to be sensible”* (female, 56 years, healthcare worker). Some feared crossing paths with strangers in shops and outdoors, for risk of contracting COVID-19: *“I felt quite anxious because I think, well I can do all the right things, but I can’t control what other people do”* (female, 63 years, retired). In this way, community tensions and raptured relationships appeared to be a key outcome of differing levels of compliance and opinions about the COVID-19 measures, which some expected would continue in the future: *“this is certainly such a new thing and people are coming to different conclusions… and [it] will be an ongoing challenge to navigate within relationships”* (female, 24 years, healthcare worker).

### Impact on wider society and politics: between polarisation and compassion

Closely related to narratives of division within communities, many participants also reflected on the wider societal implications of COVID-19 measures, saying that the pandemic *“has really polarised people’s political views… In some ways Coronavirus has brought people together, but in other ways, it’s made people more political and divided”* (male, 33 years, healthcare worker). As such, while some defended the government’s COVID-19 response – *“we locked down quite hard and I think that made sense”* (female, 24 years, healthcare worker) – others voiced anger and frustration: *“we haven’t been following the science and we should have locked down earlier and harder and that could have saved many, many lives”* (male, 55 years, IT professional). Several participants expressed a general lack of trust in the government: “ *It is very difficult to trust a lot of the people who seem to be in charge”* (female, 80 years, retired). Some linked the political polarisation in response to COVID-19 measures to the breakdown of their personal relationships: *“I have actually fallen out with a friend… We have always had different political views, but I never let it get in the way… This time I decided to cut ties with him”* (male, 33 years, healthcare worker).

Many conveyed confusion and frustration about the COVID-19 measures:

*“nothing has been clear. I think it’s been very dangerous how [the government] has dealt with it… it’s like a parent who is not there… their job really should be to keep us safe… but they are not, and we are all scrambling around trying to keep us safe and our families safe… there are so many mixed messages”* (female, 56 years, self-employed).

This lack of clarity was said to cause *“a lot of stress… from trying to interpret what they were saying… and it feels as though it’s the governments way of passing the blame onto ourselves”* (male, 39 years, social worker). This was also felt to negatively impact on community relationships: *“It fragments communities because we’re all doing our best but all with different ideas because there isn’t a clear structure”* (female, 56 years, self-employed).

Many participants drew comparisons between themselves and society at large, highlighting the differences in lived experiences of the lockdown. Discussing narratives of privilege and inequality, several participants described themselves as *“lucky”* (female, 53 years, unemployed), *“fortunate”* (male, 53 years, support worker) and *“privileged... I have work, I’m in good health, my family is safe… where I live… I can reach a huge meadow by a river”* (female, 71 years, freelance professional). The ability to keep safe from COVID-19, such as by virtue of having access to a garden or living rurally was seen as a privilege: “ *I am lucky, I live in a village sort of in the countryside, so you could quite easily go out or cycle into the countryside and you wouldn’t come across people”* (female, 46 years, finance officer).

In contrast, some said they were facing significant hardship and felt particularly disadvantaged compared to others as a result of COVID-19 measures, because of living in small flats, facing income insecurity, caregiving responsibilities, or fears about returning to the workplace:

“ *… if… they say ‘come into work’ and I'll say ‘I'm sorry but we're still on the second wave’ then I will have to face whether I lose my job... But I will not put myself in danger…. I am not dying for minimum wage, I am not”* (male, 53 years, support worker).

A few highlighted wider social and economic inequalities underpinning the impact of the lockdown, suggesting that “ *actually [the virus has] not been the great leveller that everyone suggested. It's actually highlighted where there are massive inequalities* (male, 55 years, IT professional):

*“A lot of people, people who usually get the short straw, are still getting it now. People who haven’t got secure jobs and gardens and are in high rise flats with small children… it’s the people who can't work from home who have to go back to work. And they haven't got cars a lot of them, so they have to go on the public transport which they are advised not to do… it is very depressing.”* (female, 80 years, retired).

Some discussed how this awareness of certain groups, including “ *the homeless… young people*” (female, 71 years, freelance professional) and *“BAME [Black, Asian, and minority ethnic] people suffering disproportionately”* (female, 48 years, support worker) evoked a sense of solidarity, compassion and reflections on social inequality:

*“Something that worries me about what is going to happen, particularly for the usual groups who pay the price… Because for people like me, we are going to be fine… but there is a lot of people… I’m really scared from their point of what’s going to happen.”* (female, 71 years, freelance professional).

## Discussion

This study provides unique record of the lived experience of people living through the first UK national COVID-19 lockdown, detailing the relational experiences at this time of unprecedented public health and social distancing measures. Drawing on an adapted ecological framework, the study outlines the significant impact of COVID-19 NPIs on relationship dynamics and social connections at every relational level. The sudden disruption of social dynamics and human relationships during the lockdown, which led to a “fundamental, if short-term, collapse of conventional social order” ( Strong, 1990, p. 225), forced a reconstruction of the social world – including in the relationship with oneself (self-perception); one’s household and intimate partners; family and friends; communities and neighbours; and with wider society and politics. While, for most participants this led to previously established relationships and social norms being disrupted, some also experienced greater social connectedness, (re)connection and (re)commitment to existing relationships in response to the lockdown measures.

As such, our findings resonate with early conceptualisations of fatal epidemics as “dramatic social cris[e]s” causing fear, suspicion, moralising, disruption and disorientation ( Strong, 1990, p. 250). However, they also point to another, less examined aspect of lived reality; namely the experience of greater intra- and interpersonal connection and compassion during times of health crisis, also discussed in other studies ( Abendschein *et al.*, 2021; Whitehead & Torossian, 2021). Reverberating the meaning of the Greek root of the word crisis ( *κρίσις*), meaning “discrimination, decision” ( Oxford English Dictionary, 2021), our findings show that being faced with the COVID-19 crisis caused many to reflect on, distil and decide to nurture those relationships regarded as most valuable and important.

This study builds on an increasing body of research showing that changes to relationship dynamics, in the form of relational adaptation, improvement, or deterioration have been a common experience during lockdown across countries ( Chu *et al.*, 2021; Günther-Bel *et al.*, 2020; Li & Samp, 2021; Wisyaningrum *et al.*, 2021; Wong *et al.*, 2021). These findings are important in light of growing evidence on the negative impacts of loneliness and lack of social connectedness on physical and psychological health ( Hawkley & Cacioppo, 2010; Holt-Lunstad *et al.*, 2015). In this study, experiences of disconnection, relationship tensions and breakdowns in response to COVID-19 restrictions were perceived as significant and additional source of stress and uncertainty during the lockdown.

While all participants experienced some disruptions to their relationships and social dynamics in response to the lockdown, the extent to which these were perceived as negative varied considerably. Importantly, those who were able to draw on positive household, family and community relationships as a source of practical and emotional support during lockdown, experienced their relationships as helpful for handling pandemic related stressors. Other studies have also shown that positive relationships and a sense of connectedness are important factors to support individual coping and resilience during times of stress and adversity ( Cohen, 2004), including during the COVID-19 pandemic ( Landmann & Rohmann, 2021). Similarly, research on the experience of living with COVID-19 infection highlights that caring relationships and social connections are key to feeling supported and able to cope with uncertainty and isolation ( Missel *et al.*, 2021).

Furthermore, our findings point towards the central role of trust during crises, showing that experiences of distrust of others, one’s community or neighbours, and of the UK governments’ handling of the pandemic led to social tensions and polarisation at every level. Similarly, trust in government was found to mediate the association between community resilience and anxiety during the COVID-19 pandemic ( Zhang *et al.*, 2021) and to be associated with greater compliance with COVID-19 measures ( Dohle *et al.*, 2020). Recent research on the role of social cohesion in the UK further suggests that investments in community integration programmes prior to the pandemic helped individuals and communities cope better with the impacts of COVID-19, and resulted in stronger and more connected communities, as measured by higher levels of reported social activism, closer personal relationships, and greater interpersonal and political trust ( Lalot *et al.*, 2021). Our research thus supports others who call for interventions to support trust, social connectedness, and cohesion at every societal level.

By documenting the plethora of lived experiences, as well as the important role of the quality of social connections in shaping lived experiences of the first UK national lockdown, our findings build on existing evidence that shows COVID-19 measures have given rise to multiple, anachronic and diverse lived realities within the same country ( Osterrieder *et al.*, 2021; Schneiders *et al.*, 2021). These findings lead us to suggest expanding existing definitions of COVID-19 related vulnerability from a focus solely on biological factors to also include social factors, most notably considerations about social capital, cohesion, and connectedness ( Lalot *et al.*, 2021; Pitas & Ehmer, 2020; Wu, 2021). Our study thus highlights the need to better support those most vulnerable to the negative effects of isolation during COVID-19 lockdowns, and echoes other authors calling for increased support for socially vulnerable groups, including older people ( Arpino *et al.*, 2021; van Tilburg *et al.*, 2021) and those living on their own ( Kamin *et al.*, 2021).

Furthermore, participant’s experiences of lockdown uniquely highlight the interconnectedness of the various socio-ecological systems within which they were embedded. For example, many reported experiences of the personal becoming politicised and politics becoming personal, such as when different interpretations of government rules led to conflict within interpersonal relationships. Furthermore, the impact of the lockdown on relationship dynamics as shown in this study reveals interesting parallels across the individual, interpersonal and wider societal level. While participants’ accounts indicate a wide range of lived experiences and emotions — between “fear, grief, and time for personal reflection” at the individual level; “growing closer and ruptured relationships” within households and intimate relationships; “virtual connection and longing for physical touch” within family and friendships; “solidarity and distrust” within communities and neighbourhoods; and “polarisation and compassion” within society and politics — taken together these findings expose a common theme; namely, an over-arching tension between the need for both *distance* and *connection* during the lockdown. This tension operated within and across the individual, interpersonal, and structural level, thereby resonating with ecological approaches ( Bronfenbrenner, 1979), by highlighting how the interactions and interdependence of different life domains shaped lived experiences of the first UK national lockdown.

### Study strengths and limitations

Our study has several strengths and limitations. Firstly, the use of online qualitative research methods, while they facilitated data collection during lockdown restrictions, and had benefits, such as the inclusion of a broader range or participants, also had drawbacks, including limiting both non-verbal communication and participant-researcher relationship building ( Davies *et al.*, 2020). While, for this study we were successful in purposively recruiting from a range of age groups, occupations and levels of self-perceived COVID-19 risk, due to rapid recruitment, geographical representation from across the four UK nations was limited (England: n=18; Wales: n=1; Scotland=1). Even though restrictions during the first national lockdown were uniform across the four nations, subsequent policy changes which resulted in different lockdown measures across the four UK nations warrant further research into the different lived experiences based on geography, including between the four UK nations and urban and rural residents. This study also did not gather data on participant’s ethnicity or income, which recent research has found to have a significant impact on well-being during COVID-19 lockdowns ( Chakrabarti *et al.*, 2021). Additionally, despite purposive sampling and targeted study advertisement among community organisations, most participants (75%) had completed tertiary education. The remote method of data collection using Microsoft Teams, which was the only online platform to satisfy data protection regulations, may have hindered participants with lower technological skills from taking part. While participants were given the option of a telephone interview, this may not have been sufficient to offset the study’s inability to conduct interviews via more accessible platforms like WhatsApp or Facebook Messenger. Despite these limitations, using a rapid recruitment strategy enabled unique insights into lived experiences during the early stages of the UK lockdown, a time at which few qualitative COVID-19 studies were being conducted. Finally, several participants expressed valuing the opportunity to have a safe space in which to reflect on and share their feelings on this unprecedented and disruptive time in their lives, adding to literature on the potential therapeutic value of the participant-researcher relationship and the research interview as a space for empathic witnessing and healing of difficult experiences ( Colbourne & Sque, 2005; Drury *et al.*, 2007; Priya, 2010; Rossetto, 2014).

## Conclusion

Our findings highlight the social relational disruptions that resulted from NPIs aimed at reducing close physical contact to prevent COVID-19 transmission during the first UK lockdown. This disruption of sociality impacted relationships at every level, albeit differently depending on prior social connectedness, leading to common experiences of connection but also of division. As such, lived experiences of lockdown differed in important ways depending on the quality of prior interpersonal relationships, wider social connections and the support networks within which participants were embedded. This suggests the importance of considering social connectedness as a key element of COVID-19 NPIs, to support individual and community-level resilience in light of ongoing COVID-19 restrictions. Since the first UK national lockdown, some progress has been made to this end, including through the introduction of “household bubbles” for people living alone and through increasing availability of virtual support services. Public health messaging should continue to focus on promoting interventions that maintain social connectedness and promote alternative ways of maintaining social contact in safe and effective ways in the face of ongoing NPIs.

## Data and software availability statement

### Underlying data

All relevant data are within the manuscript. Complete interview data cannot be made publicly available due to ethical and legal reasons. These reasons relate to protecting the interests of research participants, as interview transcripts cannot be de-identified without compromising anonymity, and data protection concerns, as we also do not have blanket consent to share interview data openly without restrictions. Upon reasonable request, a list of condensed meaning units or codes can be made available on request to the MORU Data Access Committee (see link https://www.tropmedres.ac/units/moru-bangkok/bioethics-engagement/data-sharing).

### Extended data

Zenodo: Topic guide for qualitative study: Social, ethical and behavioural aspects of COVID-19. https://doi.org/10.5281/zenodo.3777934 ( Poomchaichote *et al.*, 2020)

This project contains the following extended data:

- The interview topic guide

Data are available under the terms of the Creative Commons Attribution 4.0 International license (CC-BY 4.0).

## Software availability

Data was managed using the propriety qualitative data software NVivo (v12). Alternative free and open source software that can perform an equivalent function to NVivo are Taguette and RQDA.
