# Supplementary material for: Between division and connection: a qualitative study of the impact of COVID-19 restrictions on social relationships in the United Kingdom
Source: Wellcome Open Res. 2022 Jan 6;7:6. [Version 1] doi: 10.12688/wellcomeopenres.17452.1 (PMC9065928; doi:10.12688/wellcomeopenres.17452.1)
Supplement: Peer review history

# Peer review: Reviewer response for version 1

**DOI:** 10.21956/wellcomeopenres.19298.r49647

**Published:** 2022-05-03

**Authors:** Aimi Nadia Mohd Yusof

**Affiliations:** 1 Faculty of Medicine, Universiti Teknologi MARA, Sungai Buloh, Malaysia

It is with great pleasure to be given the opportunity to review this interesting piece of research. The objective of the research is to understand people’s lived experiences with the COVID-19 lockdowns in the UK, mainly the impact on social connections and relationships. This research uses a qualitative approach to interview the participants online.

 In the introduction, it is not clearly stated what comprises non-pharmaceutical interventions (NPIs) and if it is relevant to provide a scope of the NPIs that the researchers are focusing on.

 The use of a qualitative approach is appropriate to explore and understand the experiences and attitudes of the participants. Referring to participants’ characteristics, I think the range of financial income is also an important component that might affect their experiences and attitudes towards the lockdowns.

 Given the extent of the five levels of the ecological framework adapted with a sample size of 20 participants, researchers might want to add more information on how the thematic saturation is achieved.

 Overall, I find the findings and discussion relevant and important to contribute to further improvements in managing lockdowns in the future.

Is the work clearly and accurately presented and does it cite the current literature?

Yes

If applicable, is the statistical analysis and its interpretation appropriate?

Not applicable

Are all the source data underlying the results available to ensure full reproducibility?

Yes

Is the study design appropriate and is the work technically sound?

Yes

Are the conclusions drawn adequately supported by the results?

Yes

Are sufficient details of methods and analysis provided to allow replication by others?

Yes

Reviewer Expertise:

Research Ethics

I confirm that I have read this submission and believe that I have an appropriate level of expertise to confirm that it is of an acceptable scientific standard.

# Peer review: Reviewer response for version 1

**DOI:** 10.21956/wellcomeopenres.19298.r48937

**Published:** 2022-03-23

**Authors:** Jonathan Clarke

**Affiliations:** 1 Centre for Mathematics of Precision Healthcare, Imperial College London, London, UK; 2 Department of Mathematics, Imperial College London, London, UK

Thank you for giving me the opportunity to review this timely and well-written manuscript. This is a qualitative interview study exploring the experiences and attitudes of individuals in the United Kingdom to nonpharmaceutical interventions as part of the COVID-19 pandemic. It appears that the qualitative component of this study is part of a larger study known as SEBCOV. It's not clear from the paper what the overall aim of the study is and it may be helpful, rather than a reference, for the authors to explain the scope of the larger study of which this study is a part. 

 The authors openly acknowledge the role of selection bias in the recruitment of participants, particularly given that 75% of participants are university graduates. It may be useful to discuss the potential implications this may have for the generalisability of findings, if any. Similarly, it is unclear the extent to which participants live in urban or rural areas and this may influence the experience of individuals in response to lockdown restrictions.

 The use of an ecological framework in the analysis of the interviews is entirely appropriate and provides a very useful structure by which to interpret the findings. Given the breadth of the material covered in the interviews, I am surprised that meaningful saturation was achieved with a sample of 20 interviews; this may be something that the authors wish to comment upon in their discussion of findings.

 Interviews were conducted from 14 May to 23 July 2020 and although this is a relatively short period of time it did compass quite significant changes in the incidence of COVID-19 and potentially attitudes of individuals towards restrictions. It may be helpful for the authors to comment on the extent to which the time at which an interview was conducted related to individual attitudes.

 As a reader working in health policy, while the study itself provided lots of interesting insights, I was less sure of what I could do differently based on the findings of the study. Perhaps a paragraph on implications of the work for policy and practice might help readers incorporate the findings of the work into their practice. 

 Overall, this study is well conducted and written and will help provide an important insight into our understanding of the lived experience of people in the UK during the first wave of the COVID-19 pandemic.

Is the work clearly and accurately presented and does it cite the current literature?

Yes

If applicable, is the statistical analysis and its interpretation appropriate?

Not applicable

Are all the source data underlying the results available to ensure full reproducibility?

No source data required

Is the study design appropriate and is the work technically sound?

Yes

Are the conclusions drawn adequately supported by the results?

Yes

Are sufficient details of methods and analysis provided to allow replication by others?

Yes

Reviewer Expertise:

Health policy and health system responses to COVID-19.

I confirm that I have read this submission and believe that I have an appropriate level of expertise to confirm that it is of an acceptable scientific standard.
